# Human mesenchymal stromal/stem cells recruit resident pericytes and induce blood vessels maturation to repair experimental spinal cord injury in rats

**DOI:** 10.1038/s41598-020-76290-0

**Published:** 2020-11-11

**Authors:** Karla Menezes, Barbara Gomes Rosa, Catarina Freitas, Aline Silva da Cruz, Raphael de Siqueira Santos, Marcos Assis Nascimento, Daiana Vieira Lopes Alves, Martin Bonamino, Maria Isabel Rossi, Radovan Borojevic, Tatiana Coelho-Sampaio

**Affiliations:** 1 Post-Graduate Program in Morphological Sciences, Institute of Biomedical Sciences, Federal University of Rio de Janeiro, Ave. Carlos Chagas Filho 373, Sala B1-011, Cidade Universitária, Ilha Do Fundão, Rio de Janeiro, RJ 21941-590 Brazil; 2 Center for Regenerative Medicine, Petrópolis Faculty of Medicine, Av. Barão do Rio Branco 1003, Centro, Petrópolis, RJ 25680-120 Brazil; 3 Alberto Luiz Coimbra Institute for Graduate Studies and Engineering Research (COPPE), Federal University of Rio de Janeiro, Ave. Carlos Chagas Filho 373, Sala B1-011, Cidade Universitária, Ilha Do Fundão, Rio de Janeiro, RJ 21941-590 Brazil; 4 Institute of Biophysics Carlos Chagas Filho, Federal University of Rio de Janeiro, Ave. Carlos Chagas Filho 373, Sala G2-053, Cidade Universitária, Ilha Do Fundão, Rio de Janeiro, RJ 21941-590 Brazil; 5 Institute for Biodiversity and Sustainability - NUPEM, Federal University of Rio de Janeiro, Ave. São José Do Barreto S/N - São José Do Barreto, Macaé, RJ 27965-045 Brazil; 6 National Institute of Cancer, Rio de Janeiro, Marquês de Pombal, Centro, Rio de Janeiro, RJ 20230-240 Brazil

**Keywords:** Mesenchymal stem cells, Spinal cord diseases

## Abstract

Angiogenesis is considered to mediate the beneficial effects of mesenchymal cell therapy in spinal cord injury. After a moderate balloon-compression injury in rats, injections of either human adipose tissue-derived stromal/stem cells (hADSCs) or their conditioned culture media (CM-hADSC) elicited angiogenesis around the lesion site. Both therapies increased vascular density, but the presence of hADSCs in the tissue was required for the full maturation of new blood vessels. Only animals that received hADSC significantly improved their open field locomotion, assessed by the BBB score. Animals that received CM-hADSC only, presented haemorrhagic areas and lack pericytes. Proteomic analyses of human angiogenesis-related factors produced by hADSCs showed that both pro- and anti-angiogenic factors were produced by hADSCs in vitro, but only those related to vessel maturation were detectable in vivo. hADSCs produced PDGF-AA only after insertion into the injured spinal cord. hADSCs attracted resident pericytes expressing NG2, α-SMA, PDGF-Rβ and nestin to the lesion, potentially contributing to blood vessel maturation. We conclude that the presence of hADSCs in the injured spinal cord is essential for tissue repair.

## Introduction

Spinal cord injury (SCI) is a major cause of physical disability in young adults^1^. Although SCI has attracted intense research efforts, efficient treatments have not been clinically validated so far. Induction of post-injury angiogenesis is essential for effective tissue repair, and targeting vascularization is a potentially valuable therapeutic strategy for SCI^2^.

Regeneration of a hierarchically ordered and mature vascular network depends upon interactions of different cells, and is mediated by several growth factors^3^. After SCI vascular sprouts and immature capillaries emerge from the neighbouring blood vessels, indicating that vascular regeneration in the central nervous tissue begins from the pre-existing vessels^4^. Within the first seven days after injury, a rapid increase in angiogenesis occurs at the lesion site^5^. Subsequently, the new vascular network has to undergo extensive remodelling to form a functional vasculature^2,3,5^.

Pericytes play a fundamental role during vascular stabilization and maintenance of the blood–brain barrier^6^. Resident pericytes also participate in the formation of a fibrotic scar tissue, and contribute to the endogenous tissue repair after SCI^7^. They proliferate, migrate to the lesion area, and differentiate into ECM producing fibroblasts^8^. Pericytes have even been proposed to acquire multipotency after a severe encephalic ischemia and to contribute to neurogenesis^9^.

In regenerative medicine, the use of adipose tissue-derived stromal/stem cells (ADSCs) is a promising strategy to promote angiogenesis. The benefits of ADSCs transplantation have been previously demonstrated by the functional recovery of animals submitted to experimental SCI^10–23^. Although the results in humans are still preliminary, autologous ADSCs transplantation was considered safe^24^, and it promoted motor and somatosensorial improvement after spinal cord trauma^25^.

The beneficial effects of hADSCs in central nervous system (CNS) regeneration are thought to result from their paracrine activity^26^. ADSCs can secrete high amounts of angiogenic factors, such as vascular endothelial growth factor (VEGF), fibroblast growth factor (FGF), platelet-derived growth factors (PDGFs), insulin-like growth factor 1 (IGF-1) and hepatocyte growth factor (HGF)^27–29^. In addition, mesenchymal cells have been shown to secrete exosomes carrying microRNAs that can be uptaken by endothelial cells, stimulating tube-formation^30^. While it has been extensively shown that hADSCs conditioned medium contains factors capable of inducing angiogenesis in in vitro assays^27,28,31,32^, it is not yet known whether these paracrine mediators are sufficient to promote vascular repair after CNS injury in vivo.

Here we compared the therapeutic use of soluble factors produced by hADSCs in vitro with transplantation of live cells after SCI. The potential advantage of soluble factors would be that the supply of in vitro-produced bioproducts could be scaled-up, stored and subsequently offered “from shelf”. We found evidence that hADSCs transplanted into the spinal cord modulate their secretion and activity of angiogenic factors in response to the environment and, hence, could not be replaced by conditioned medium produced in vitro. Furthermore, engrafted hADSCs contribute to maturation of the newly formed vasculature through attraction of endogenous pericytes, selectively favouring the functional recovery of animals that received the cell therapy.

## Results

### hADSCs and CM-hADSC increased the number of blood vessels at the perilesional region after a compressive spinal cord injury.

**Figure 1:**
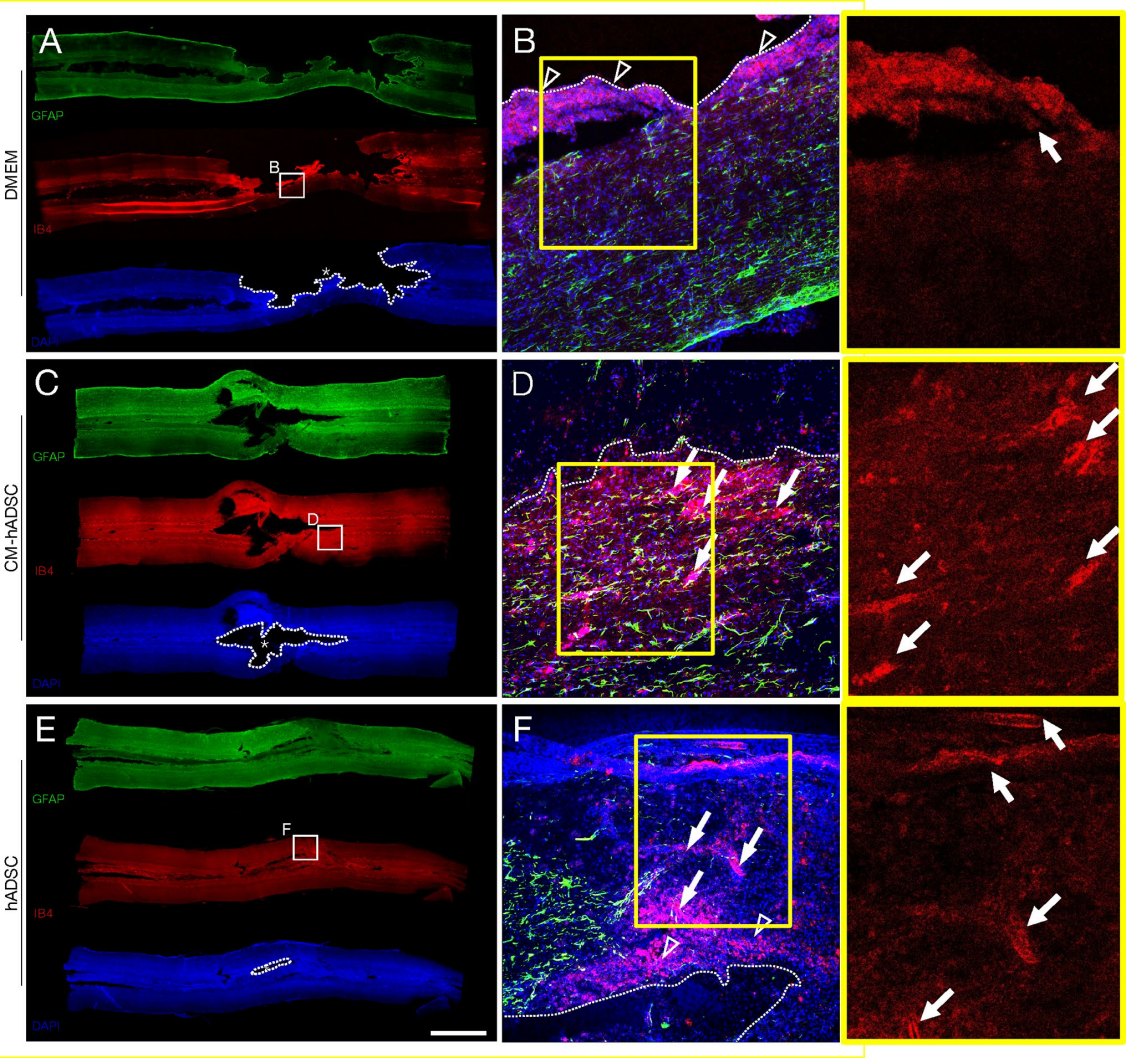
*hADSCs and CM-hADSC increased the number of blood vessels at the edges of lesion cavities after a compressive spinal cord injury*. Low-magnification micrograph photomontages of the spinal cord (horizontal sections, approximately 500 -750 μm down from the dorsal surface, identified as the lesion epicentre by GFAP reactivity) of rats treated with DMEM (**A**), CM-hADSC (**C**) and hADSC (**E**), 1 week after SCI, showing blood vessels and phagocytic cells, identified with Isolectin IB4 (red, **A**, **C**, **E**), and its interaction with astrocytes, identified with anti-GFAP (green, **A**, **C**, **E**), and DAPI-stained nuclei (blue, **A**, **C**, **E**). Confocal images under high magnification of the boxed fields show the nervous tissue at the edges of lesion cavities (**B**, **D**, **F**). Areas marked by in yellow boxes were further amplified and shown in the red channel only to better reveal the morphologies of blood vessels. White arrows indicate blood vessels and white arrowheads indicate phagocytic cells (red round cells). White thick dotted lines delineate lesion cavities. Scalebar: **A**, **C**, **E** 3 mm; and **B**, **D**, **F** 200 μm .

Mechanical trauma caused by a dorsal balloon compression of the spinal cord leads to rupture of local blood vessels, haemorrhage and tissue oedema. We sought to identify differences in vascular density 1 week post-injury (WPI), using Isolectin IB4 from *Griffonia simplicifolia* that labels rat endothelial cells, macrophages and microglia, simultaneously revealing vascular profiles and phagocytic cells, which are clearly distinguishable by their morphology (Supplementary Fig. S1). Analyses were performed in the tissue sections bearing the largest cavities (approximately 500–750 μm down from the dorsal surface), which were identified by astrocyte reactivity using GFAP antibody. Control animals receiving only unconditioned DMEM presented a larger lesion cavity devoid of tissue as shown by the complete lack of DAPI, GFAP or IB4 staining (Fig. 1A). The inset represents a higher magnification image to allow identification of glial cells (green GFAP + cells in Fig. 1B) and massive phagocyte infiltration (red IB4 + round cells in Fig. 1B, arrowheads) at the edges of the lesion cavity, while no vascular structures could be identified (Fig. 1B). Animals that received hADSCs exhibited a distinct pattern of tissue repair (Fig. 1E). Dapi and IB4-stainings indicate that the cavity had been partially filled with a cell-dense tissue (Fig. 1E,F), containing macrophages/microglia and abundant blood vessels (identified by their morphologies; Fig. 1F, white arrows), presenting weak GFAP-labeling (Fig. 1E,F). Higher magnification image at the edge of the cavity also revealed the presence of IB4 + blood vessels (Fig. 1F, arrowheads). Treatment with CM-hADSC did not lead to formation of a cavity-filling cellularized tissue (Fig. 1C), therefore, it was not possible to compare vascular densities within the lesion epicentre. Interestingly, we observed the presence of an increased number of blood vessels at the cavity edges in animals receiving CM than in animals receiving cells (white arrows, Fig. 1D,F and corresponding amplifications within yellow squares).

**Figure 2:**
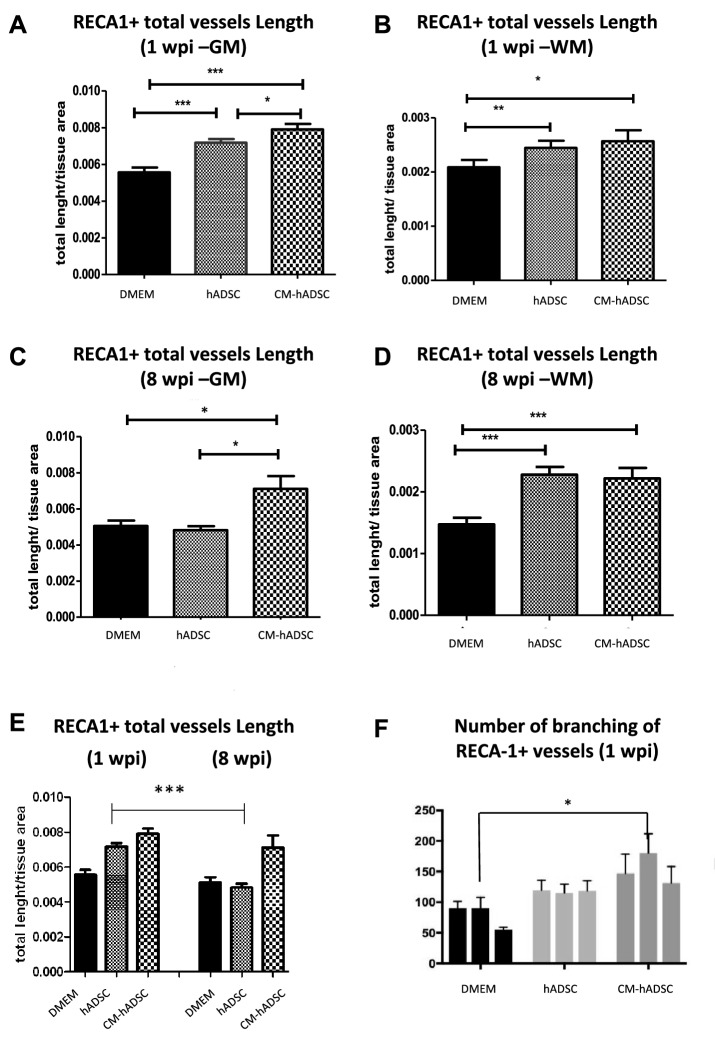
Treatment with CM-hADSC and hADSC promoted increased vascular length in relation to control DMEM animals. (**A**–**D**) Quantification of the total length of RECA-1 immunostained blood vessels per tissue area at the grey (GM) (**A**, **C**) or white matters (WM) (**B**, **D**), in the perilesional region, 1 week (**A**, **B**) and 8 weeks (**C**, **D**) after injury. (**E**) Graph representing the number of blood vessels in the acute and chronic phases of SCI. (**F**) Graph representing vessel junction density, indicating the number of branching points of RECA-1 vessels. All quantitative analyses evaluated 3 animals per experimental group, and were performed in horizontal sections. In all graphs the first, second and third columns refer to DMEM, hADSC and CM-hADSC animals, respectively. Values in graphs represent the means ± standard errors. *P < 0.05; **P < 0.01; ***P < 0.001.

**Figure 3:**
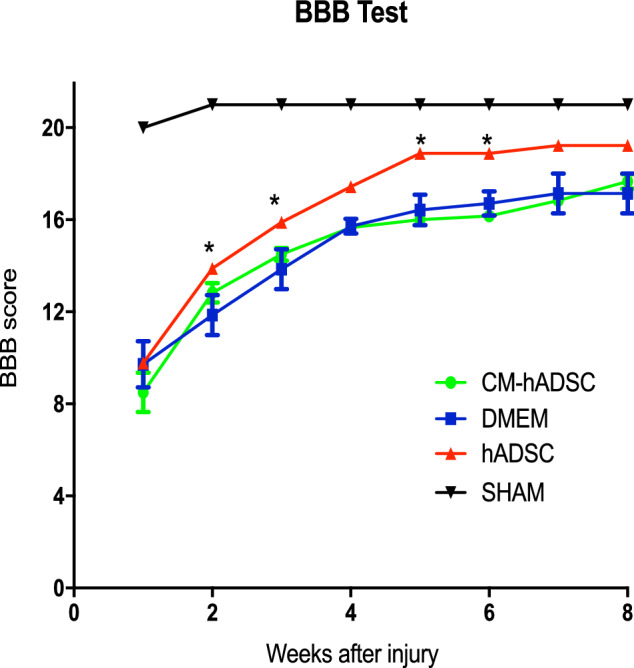
Treatment with hADSC, but not CM-hADSC, promoted functional recovery after spinal cord compression. Graph representing the locomotor performance, which was assessed by the BBB score. Animals were evaluated weekly, during eight weeks after spinal compression. The curves described in the graph in black, blue, green and red line represent animals from the experimental groups SHAM (surgical control, without laminectomy), DMEM, treated with CM-hADSC and hADSC, respectively. Values in graphs represent the means ± standard errors. *P < 0.05; **P < 0.01; ***P < 0.001.

In order to quantify blood vessels appearing in each experimental group, we chose to assess the tissue at the perilesional region, which we defined as the area of preserved tissue in the section containing the largest area of cavitation. We used the RECA-1 antibody, which reacts with a rat endothelial cell antigen. At 1 WPI, animals that received hADSCs or CM-hADSC had higher vascular density, as measured by total vessel length, in both grey and white matters, compared to the control group (Fig. 2A,B). The vascular density in the grey matter of rats in the CM-hADSC group was significantly higher than the density in animals that received hADSC, consistent with the abundance of blood vessels observed in Fig. 1J. At 8 WPI, animals treated with hADSCs had as many blood vessels in the grey matter as animals that received DMEM, while those treated with CM-hADSC exhibited a higher vascular density in the perilesional region (Fig. 2C). Both treatments led to an increase in blood vessel length in the white matter, compared to the control group (Fig. 3D). When data in Fig. 2C,D were plotted together at the two times points, it became visible that cumulative blood vessel length in the perilesional area of animals receiving hADSCs regressed in the chronic phase of the injury, while in animals receiving CM-hADSC it did not (Fig. 2E). We have also quantified the number of branching points in the vasculature around the cavity as a reporter of sprouting angiogenesis. Although statistical difference relative to control was found only for the CM-hADSC, a tendency (P < 0.05) to increased branching was detected with both treatments (Fig. 2F).

### CM-hADSC treatment did not promote functional recovery after SCI

In order to compare the functional outcome after compressive SCI in each experimental group, we monitored open field locomotion, using the BBB score^33^ (Fig. 3). Rats subjected to a moderate compression received hADSC, CM-hADSC or DMEM by intraspinal injection, 30 min after injury. Following injury and intraspinal injection, rats were weekly evaluated for 8 weeks. BBB scores for the CM-hADSC group were indistinguishable from the DMEM control group throughout the analysed period. Conversely, animals treated with hADSCs exhibited superior scores from the first evaluation on, and higher scores became statistically significant (P < 0.05) from the second week on. This result indicated that a denser vascular network at the periphery of the lesion in CM-hADSC-treated animals did not correlate with functional improvement.

### hADSCs favoured maturation of the blood brain barrier

**Figure 4:**
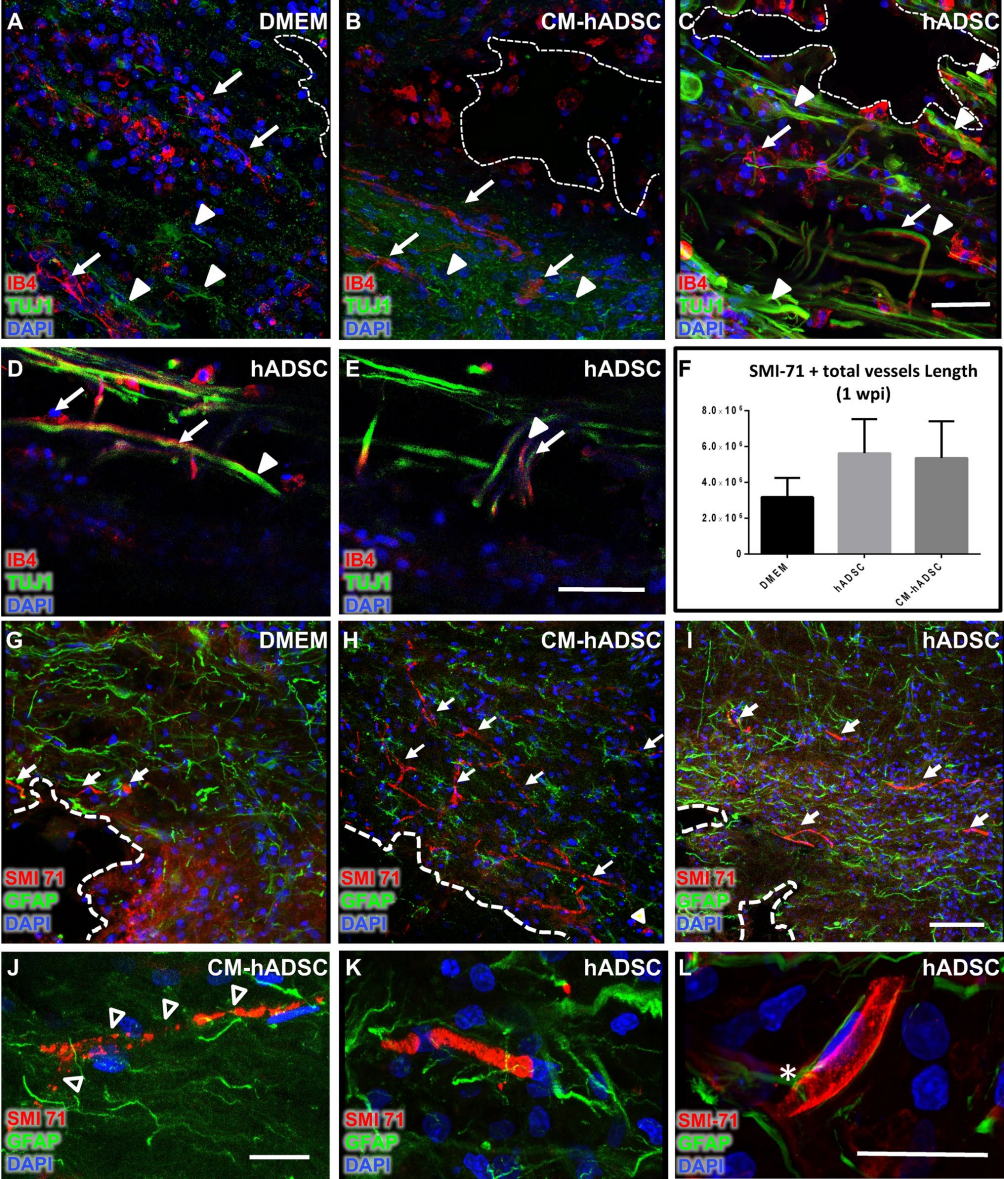
hADSCs favoured maturation of the blood brain barrier. (**A**–**C**) Confocal images of the spinal cord (horizontal sections) of rats treated with DMEM (**A**), CM-hADSC (**B**) and hADSC (**C**), labelled with TUJ-1 (green, white-filled arrowheads), Isolectin IB4 (red, white arrows) and DAPI (blue), which recognizes young axons, endothelial and phagocytic cells, respectively. (**D**, **E**) High magnification confocal images of TUJ-1 immuno-labelled axonal fibers (green) growing in close contact with blood vessels (red) in hADSC treatment. Note that some axonal fibers were projected into the injury cavity (**E**, white arrow). (**F**) Quantification of total length of blood vessels per tissue area (immuno-labelled with specific blood brain barrier antibody^34^ anti-SMI-71), 1 WPI. (**G**, **H**, **I**) Confocal images of the spinal cord (horizontal sections) of rats treated with DMEM (**G**), CM-hADSC (**H**) and hADSC (**I**), showing blood vessels identified with the blood brain barrier protein, SMI-71 (red, white arrows), and astrocytes, identified with anti-GFAP (green), and DAPI-stained nucleus (blue). (**J**, **K**) High magnification confocal images show that in CM-hADSC animals the BSB protein SMI71 pattern is punctuated (**J**, white arrowheads), while it is continuous in hADSC–treated animals (**K**). (**L**) High resolution confocal image showing the close contact and interaction between GFAP-astrocyte (green) and the blood brain barrier (red) positive cells (white asterisk). White-filled arrowheads indicate axonal fibers (TUJ-1 positive), white arrows indicate blood vessels (anti-SMI-71 or IB4 positive), arrowheads with white outline indicate vascular breakpoints, white asterisk indicates astrocyte-blood vessel interaction, and thick white dotted lines delineate lesion cavities. Black asterisks in graphs indicate statistical differences between DMEM (n = 10), CM-hADSC (n = 10) and hADSC groups (n = 10). Values in graphs represent the means ± standard errors. *P < 0.05; **P < 0.01; ***P < 0.001. Scalebar: **C**, **E**, **I**—50 μm; **J**, **L**—20 μm.

Revascularisation of nervous tissue is an essential step for axonal regeneration^2^. The inability to recover motor functions of animals treated with CM-hADSC prompted us to examine in more detail the morphological features of blood vessels at the edges of the lesion cavity, their interaction with regenerating axons and the integrity of the blood spinal barrier (BSB). Analyses using TUJ-1, an antibody that preferentially recognizes young axons, showed few green TUJ-1 + fibres in DMEM (Fig. 4A, white arrowheads) and in CM-hADSC-treated animals (Fig. 4B, white arrowheads). On the other hand, fibres were widely distributed in the hADSC-treated group (Fig. 4C, white arrowheads), where some seem to grow in close association with IB4 + vascular tubes (Fig. 4C–E). Labelling with SMI-71, an antibody that recognizes endothelial cells in mature blood brain barrier^34^, revealed that both hADSCs and CM-hADSC favoured barrier preservation (Fig. 4F–I), although quantification of vessels labelled with SMI-71 in the perilesional region failed to demonstrate a statistically significant increase of vessel density in the treated groups relative to the DMEM control (Fig. 4F). One important observation was that SMI-71 labelling in CM-hADSC was punctuated, providing a patchy pattern (Fig. 4J). On the other hand, in the hADSC group, SMI-71 staining was continuous (Fig. 4K), which suggests that the presence of hADSC cells favours barrier integrity. Furthermore, we also found evidence of extensions of star-shaped astrocytes in hADSC animals in contact with blood vessels, indicating BSB integrity (Fig. 4L, asterisk). Interestingly, hADSC also increased SMI-71 + vessels in late spinal cord injury (Supplementary Fig. S4).

### Blood vessels in animals that received CM-hADSC were associated to areas of haemorrhage

**Figure 5:**
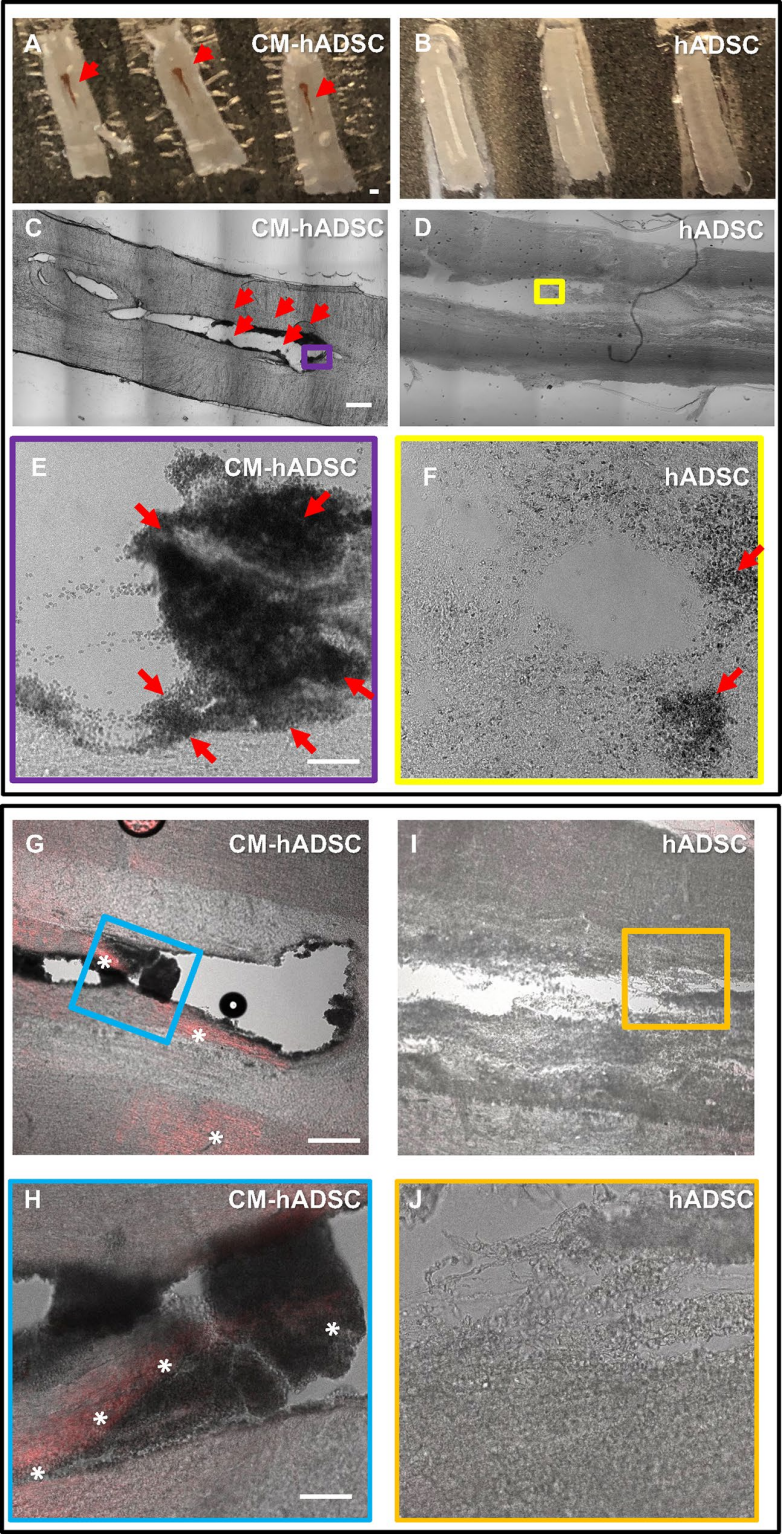
Vascular immaturity such as bleeding, plasma leakage and fenestrations in CM-hADSC-treated SCI animals. (**A**, **B**) Macroscopic images of fresh horizontal sections of rats treated with CM-hADSC (**A**) or hADSC (**B**), 1 WPI. (**C**, **D**) Confocal bright-field reconstructions of fresh horizontal sections at the lesion epicentre (approximately 250–500 μm down from the dorsal surface) of rats treated with CM-hADSC (**C**) or hADSC (**D**), 1WPI, showing hemorrhagic areas only in CM-hADSC (**A**, **C**, red arrows). (**E**, **F**) High magnification images of the boxed areas in **C** and **D**, respectively, where red arrows point to a large amount of red blood cells in CM-hADSC animals (**E**), and substantially less in hADSC—treated animals (**F**). (**G**, **I**) Confocal superimposed bright-field and fluorescence images of histological sections from CM-hADSC (**G**) and hADSC-treated animals (**I**), 1 WPI, which were stained with fluorescent anti-rat IgG (red) to detect IgG extravasation. (**H**, **J**) High magnification images of the boxed areas in **G** and **I**, demonstrated plasma extravasation (red, white asterisk) close to the hemorrhagic region in CM-hADSC animals (H) but not in hADSC treated animals (**J**). Scalebar: **A**, **C**: 500 μm, E, H: 50 μm; G: 200 μm.

Extemporaneous analyses of fresh tissue sections revealed the presence of red blood cells along the central axis of the spinal cord in CM-hADSC suggestive of increased haemorrhage (Fig. 5A, red arrows). Red blood cells were not observed in animals receiving hADSCs when inspected across sections 150 μm apart (Fig. 5B). Brightfield microscopy confirmed the abundance of red blood cells trapped at the edges of the cavity in CM-hADSC (Fig. 5C, red arrows), whereas this was not visible in hADSCs (Fig. 5D). Higher magnifications (purple and yellow boxes from panels 5C,D) showed large clusters in CM-hADSC-treated animals (Fig. 5E, red arrows) although some red blood cells were present in hADSC-treated animals (Fig. 5F). In order to confirm blood leakage, we incubated cord sections with fluorescent anti-rat IgG (Fig. 5G–J). Confocal fluorescence superimposed to bright field images showed blood leakage only in the CM-hADSC animals (Fig. 5G,H, asterisks), particularly in areas with a high density of red blood cells (Fig. 5H), whereas no IgG extravasation was detected in hADSC-treated sections (Fig. 5I,J).

### Pericytes associated with endothelial cells only in blood vessels of the hADSC-treated animals

**Figure 6:**
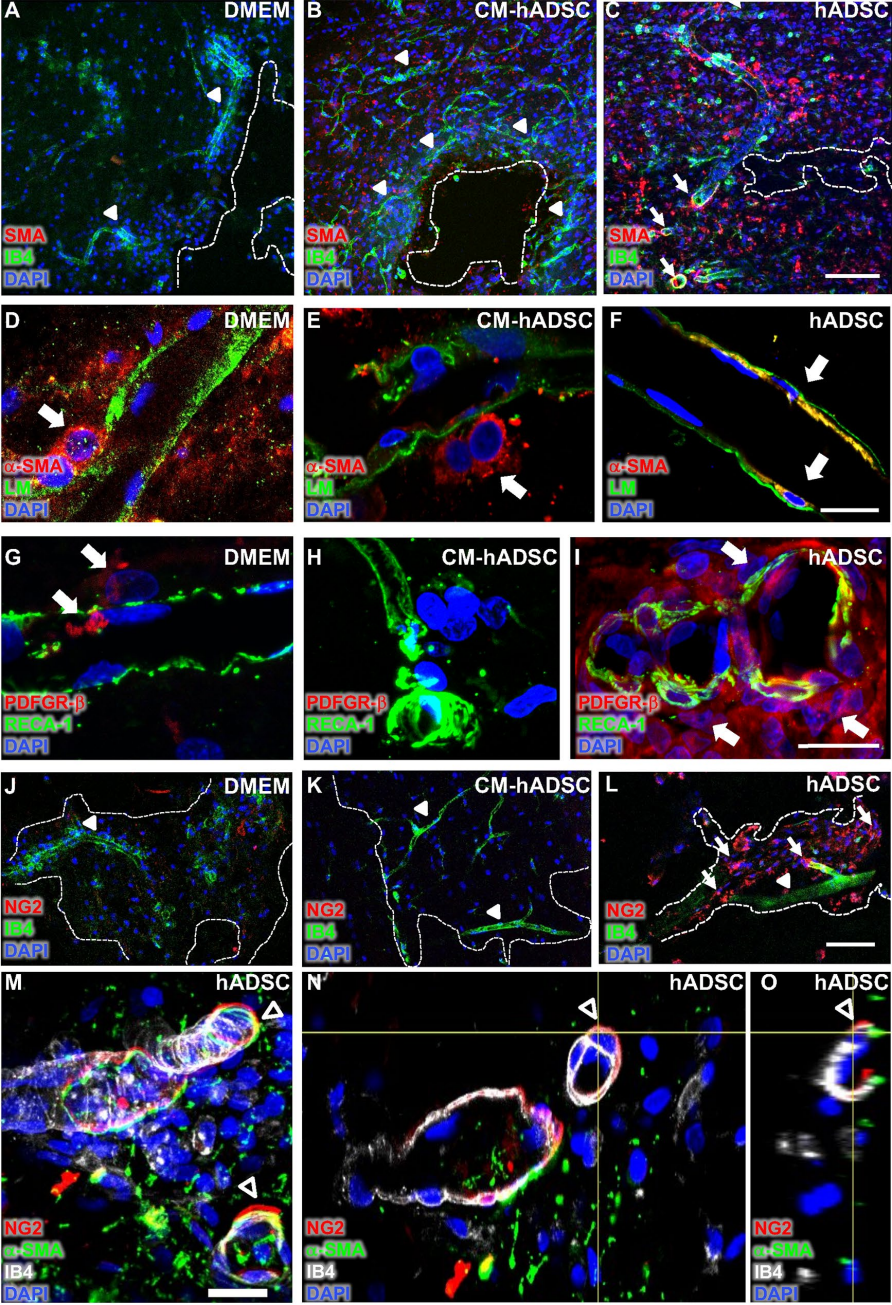
Pericytes were distributed along the blood vessels and in close contact with the vascular wall only in hADSCs treated animals. Confocal images of the spinal cord (horizontal sections) of rats treated with DMEM (**A**, **D**, **G**, **J**), CM-hADSC (**B**, **E**, **H**, **K**) and hADSC (**C**, **F**, **I**, **L**, **M**, **N**, **O**), 1WPI. (**A**, **B**, **C**) Histological sections were labelled with anti-alpha smooth muscle actin antibody (red, α-SMA), Isolectin IB4 (green, IB4) and DAPI (blue), which recognize pericytes, endothelial and phagocyte cells and nuclei, respectively. (**D**, **E**, **F**) Histological sections were immuno-labelled with pan-laminin antibody (in green), alpha smooth muscle actin antibody (red, α-SMA,) and DAPI (blue), to identify the basement membrane of the blood vessels, pericytes and nuclei, respectively. (**G**, **H**, **I**) Alternative pericyte marker, platelet-derived growth factor receptor-beta antibody (in red, PDGFR-β) and RECA-1 (green) blood vessel immunolabeling was performed to identify pericytes and endothelial cells, respectively. (**J**, **K**, **L**) Histological sections were labelled with neural/glial antigen 2 antibody (red, NG2), Isolectin IB4 (green, IB4) and DAPI (blue), to identify pericytes, endothelial and phagocytic cells and nuclei, respectively. (**M**, **N**, **O**) To certify that these vessel-associated cells were pericytes, we performed a triple staining: (1) anti-neural/glial antigen 2 (anti-NG2, red); (2) anti-α-SMA (green) and (3) Isolectin IB4 (white). The first two are considered reliable pericyte markers (anti-NG2 and/or anti-α-SMA), and the latter (IB4) identifies endothelial cells. The orthogonal view of the optical slice of confocal microscopy demonstrated the positive colocalization between anti-NG2 and anti-α-SMA (**N**, **O**). Arrowhead with white fill indicates blood vessels (IB4 positive), white arrows indicate pericytes (α-SMA, NG2, or PDGFR-β positive), arrowheads with white outline indicate close association between pericyte markers (anti-NG2 and/or anti α-SMA) and white dotted lines delineate lesion cavities. Scalebar: C, L: 100 μm, F, I, M: 20 μm.

Recruitment of pericytes is a key step in blood vessel maturation^6^. We used three different pericyte markers to better understand their relationship with nascent blood vessels. At the borders of lesion cavities, cells expressing alpha smooth muscle actin (α-SMA) were rare in the DMEM group (Fig. 6A, white arrowheads). In CM-hADSC treated animals α-SMA + cells could be detected at the cavity borders, but they did not interact with blood vessels (Fig. 6B, white arrowheads). In both groups, DMEM and CM-hADSC, the few α-SMA + cells identified in the tissue exhibited an unusual round shape and did not wrap around the vascular wall (Fig. 6D,E, white arrows). Conversely, in the hADSC animals we found a high density of α-SMA + cells, some of them associated to blood vessels (Fig. 6C, white arrows). Double labelling with the basement membrane marker laminin, highlighted their close contact with the vascular wall and embedment within the vascular basement membranes (Fig. 6F, white arrows). Detection of another pericyte marker, PDGFR-β, also confirmed the absence of pericytes covering the vascular wall of DMEM (Fig. 6G, white arrows) and CM-hADSC-treated animals (Fig. 6H), while we identified numerous cells expressing PDGFR-β surrounding blood vessels in the hADSC group (Fig. 6I, white arrows). Using NG2 as a third pericyte marker, we found clusters of NG2+ putative pericytes around blood vessels at the cavity edges in animals receiving cells (Fig. 6L, white arrows), but not in DMEM or CM-hADSC groups (Fig. 6J,K). In confocal orthogonal projections we observed that, when present, NG2-positive cells were distant from endothelial cells in animals that received CM-hADSC, while they were closely associated with the vascular wall in animals receiving hADSCs (Supplementary Fig. S2). To confirm that vessel-associated cells were pericytes, we performed a triple immunostaining using two pericyte markers (NG2 and α-SMA) and IB4 to identify endothelial cells. We found areas of positive immune co-localization for NG2 and α-SMA in vascular wall-associated cells, confirming the presence of pericytes associated to vessels of animals receiving hADSCs (arrowheads in Fig. 6M). Red (NG2) and green (α-SMA) stainings, although in very close association, were not necessarily co-localized, what can be more clearly appreciated in the orthogonal view of the confocal stack (Fig. 6N, arrowheads). Incomplete co-localization can be attributed to the fact that NG2 and α-SMA are extra and intracellularly located, respectively.

### Angiogenic factors produced in vivo by hADSCs did not match those released in vitro into the conditioned medium

Results indicate that hADSCs cultivated in vitro released factors capable of inducing the formation of new vascular tubes that did not become fully operational. In contrast, transplantation of hADSCs into the lesioned spinal cord induced the formation of mature blood vessels. We thus hypothesized that hADSCs had a distinct profile of secreted molecules once transplanted to the injured spinal cord, releasing intercellular mediators and/or structural elements appropriate to support full blood vessel maturation. To test this hypothesis, we compared 3 experimental conditions: (I) “hADSC in vitro”: the components produced by hADSCs in vitro, present in the culture supernatant, (II) “CM-hADSC in vivo”: the components extracted from tissues of rats submitted to spinal cord lesion, which had received the injection of the conditioned medium of cultured hADSCs*,* and (III) “hADSCs in vivo”: the components extracted from tissues of rats submitted to spinal cord lesion, which had received hADSCs injection. We used a species-specific human protein detection assay. Spinal tissue samples from experimental animals were harvested from the lesion epicentre 24 h after SCI.

**Figure 7:**
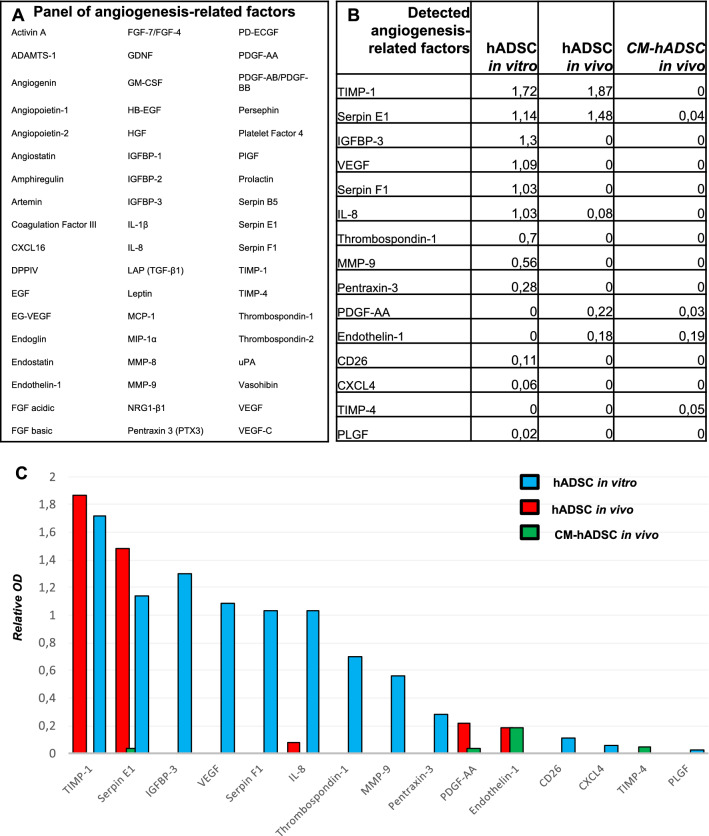
hADSCs altered the secretion of soluble pro-angiogenic factors when transplanted into the injured nervous tissue. (**A**) Table describing the 55 angiogenesis-related human proteins contained in the human-specific angiogenesis array. (**B**) Table reporting the semi-quantification of angiogenesis-related factors (first column) produced by hADSCs either in culture (“hADSCs in vitro”, second column) or in the rat spinal which had received the injection of the hADSCs (“hADSCs in vivo”, third column*)* or conditioned medium of cultured hADSCs (“CM-hADSC in vivo”, fourth column), after normalization relative to the positive control of the assay. C) Graph representing the relative concentrations of angiogenesis-related factors secreted in vitro (blue column) or in vivo, in hADSCs-treated animals (red column) or CM-hADSC-treated animals (green column). Note that the profile of angiogenic factors detected in vitro and in vivo are largely different.

Panel A in the Fig. 7 lists the 55 angiogenesis-related human proteins included in the human angiogenesis protein blot array and panel B indicates the detected proteins. The graphical representation in Fig. 7C gives semi quantitative information about the levels of the detected factors in supernatants of hADSCs cultivated in vitro and in the tissue extracted from animals injected with CM-hADSC or hADSCs after SCI. Two factors, FGF acidic and basic, were excluded from the list of detected factors because, although they appeared as positive spots in the three experimental conditions, we also found strong positivity in tissue extracted from control animals that received only unconditioned DMEM. This indicates that there was cross-reactivity between species for aFGF and bFGF. No other factors were detected in animals receiving plain DMEM (not shown), which supports the high species specificity of the assay. hADSCs secreted large amounts of angiogenesis-related factors in vitro, including proangiogenic molecules, such as VEGF, IGFBP3, IL-8, Serpin F1 and MMP9, and angiogenesis regulators such as TIMP-1, Serpin-1 and Trombospondin-1 (second column in Fig. 7B, blue bars in Fig. 7C). Analysis of spinal cord tissue extracts obtained from animals that had received hADSCs revealed a dramatic difference between the factors found in vitro and in vivo. Most proangiogenic factors, including VEGF, that were produced in vitro were not detectable in vivo, and only molecules involved in angiogenesis regulation were present in the tissue (third column in Fig. 7B and red bars in Fig. 7C). The two main factors found in vivo were TIMP-1 and Serpine-1, which are important for vasculature stabilization and maturation (Fig. 7C). Moreover, PDGF-AA that was not detected in the hADSC supernatant in vitro was present in vivo. This factor is involved in mural cells attraction, which may additionally contribute to vascular maturation. In animals that received CM-hADSC, only Serpin-E1, TIMP-4, Endothelin and PDGF-AA were detected (fourth column in Fig. 7B and green bars in Fig. 7C).

### hADSCs migrated to the lesion and attracted endogenous pericytes

**Figure 8:**
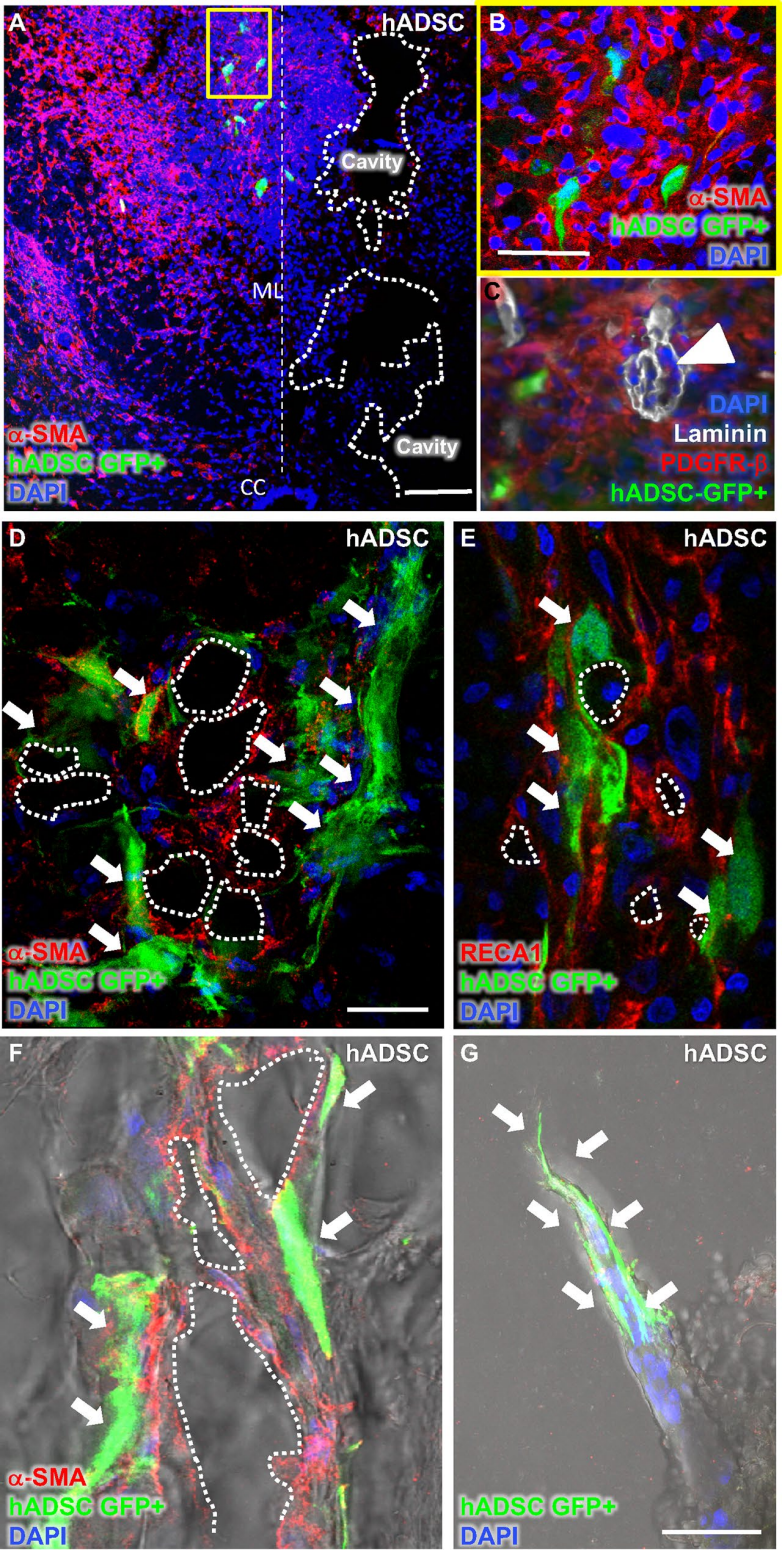
hADSCs attracted endogenous pericytes to the lesion site but did not differentiate directly into pericytes or endothelial cells. (**A**) Confocal images of transverse sections of the spinal cord, 1WPI, with GFP-transduced hADSCs (green). The nervous tissue was immuno-labelled with alpha smooth muscle actin antibody (anti-α-SMA) to identify pericytes (red) and counterstained with DAPI for cell nuclei (blue). Note that GFP + hADSCs accumulated predominantly in the central canal and in perilesional area. (**B**) High magnification confocal image of the yellow boxed area in A, where GFP + positive cell (green) clusters were seen surrounded by α-SMA positive cells (red), suggesting that hADSCs can attract pericytes (red, α-SMA). (**C**) Confocal image of the spinal cord of the animal treated with GFP + hADSCs (green) immuno-labelled with anti-pan-laminin (white), to identify the vascular basement membrane, anti-platelet derived growth factor receptor-β (PDGFR-β, red), a pericyte marker, and counterstained with DAPI for cell nuclei (blue). White arrowhead indicates PDGFR-β positive cells inside the perivascular space, between endothelial and astrocytic basement membranes. (**D**, **E**, **F**, **G**). Confocal images of a transverse (**D**) and a horizontal section (**E**, **F**, **G**) of the spinal cord from GFP + hADSC (green) animals immuno-labelled with -α-SMA (red, **D**, **F**), RECA-1 antibody (red, **E**) and counterstained with DAPI, to identify pericytes, endothelial cells and cell nuclei (blue), respectively. (**G**) Confocal image of a vascular structure, where a GFP + hADSC (green) is seen at the tip of the sprout. In images in **F** and **G** the histological sections were visualized in confocal microscopy superimposed with bright field. White arrows indicate GFP + hADSCs (**D**, **E**, **F**). CC indicates the central canal and MD, the spinal midline. White dashed lines indicate the midline of the spinal cord and thick white dotted lines delineate lesion cavities (**A**) or lumens of blood vessels (**D**, **F**, **G**). Scalebar: **A**: 100 μm; **B**–**G**: 50 μm.

In view of the role of PDGF in recruitment of pericytes, we investigated whether transplanted hADSCs would attract endogenous cells that could differentiate into pericytes for the nascent vasculature. We used GFP-transduced hADSCs to monitor the implanted cells. At 1 WPI, we observed that GFP-transduced hADSCs appeared dispersed in the spinal parenchyma, surrounded by densely packed GFP-negative cells expressing α-SMA (Fig. 8A,B) and platelet-derived growth factor receptor-β (PDGFR-β) (Fig. 8C). We found that GFP + hADCSs themselves did not express α-SMA (Fig. 8B,D,F; Supplementary Fig. S3) or PDGFR-β (Fig. 8C) within the spinal cord, although the expression of both these markers have been reported to occur in vitro upon mesenchymal cell cultivation^35^. The fact that GFP + cells were not found embedded at blood vessels, together with the fact that there were no co-localization between GFP and α-SMA or PDGFR-β, indicated that the injected cells did not differentiate into pericytes. In addition, they did not express RECA-1, which demonstrates that they neither differentiate into endothelial cells (Fig. 8E). Interestingly, GFP + hADSCs were seen in tissue foci, where cell clusters gathered around small cavities resembling blood vessel lumens (Fig. 8F, white arrows), whereas GFP + cells tended to localize at the periphery of these clusters. In some cases, injected GFP + hADSCs were incorporated into the tip of vascular sprouts (Fig. 8G, white arrows). These results suggest that transplanted hADSCs may contribute to angiogenesis by both local secretion of paracrine factors and migration towards the nascent blood vessels, as a perivascular cell population, supporting their stabilization.

### Resident pericytes attracted by hADSC to the lesion site were proliferative and expressed nestin

**Figure 9:**
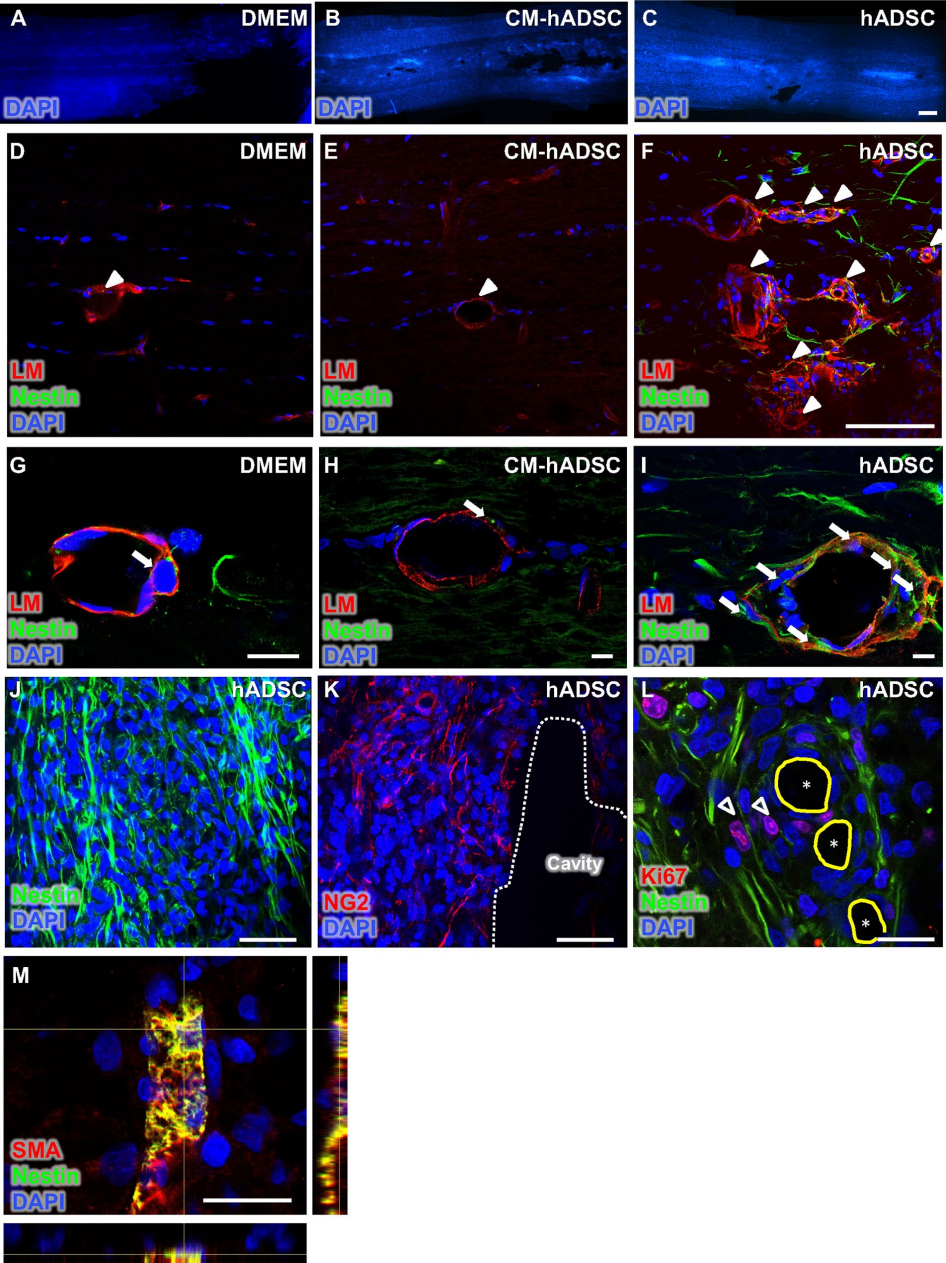
hADSCs attracted nestin positive cells, possibly expressed by resident pericytes that migrated along the perivascular space. (**A**–**C**) Fluorescence microscopy images of the spinal cord (horizontal sections) of rats treated with DMEM (**A**), CM-hADSC (**B**), hADSC (**C**), counterstained with DAPI for cell nuclei (blue), 1WPI. (**D**–**I**) Confocal images of horizontal sections of rats treated with DMEM (**D**, **G**), CM-hADSC (**E**, **H**), hADSC (**F**, **I**), immuno-labelled with anti-nestin (green), anti-pan-laminin (red) and counterstained with DAPI (blue) to identify neural precursor cells, the basal lamina of blood vessels and cell nuclei, respectively. The white arrowheads indicate the separation between the two basal laminas of the blood vessels and the white arrows indicate the presence of nestin positive cells. (**J**–**L**) Confocal images of consecutive spinal cord sections of hADSC-treated animals, 1WPI, immuno-labelled with anti-nestin, anti-NG2, anti-Ki67 and counterstained with DAPI, respectively, to identify neural precursor cells (green), pericytes (red), proliferative activity (red), and cell nuclei (blue), respectively. Note the presence of nestin positive cells that are proliferating in the perivascular region (**L**). (**M**) To certify that these pericytes express nestin, we performed a double immunostaining: (1) anti-nestin (green); (2) anti-α-SMA (red), and DAPI-stained nucleus (blue). Note that there is a positive colocalization for anti-nestin and anti-α-SMA immunostaining, suggesting that pericytes after SCI express nestin. The asterisks mark lumens of blood vessels, arrowhead with white outline indicates nestin/Ki67 double positive cells. White dotted lines delimit cavity areas, and the continuous trace in yellow indicates the lumens of blood vessels. Scalebar: **C**: 500 μm; **F**, **J**, **K**: 100 μm; **G**, **H**, **I**: 10 μm; **L**, **M**: 20 μm.

In a previous study, we showed that the spinal cord of hADSC-treated animals presented clusters of endogenous cells with proliferative capacity and a clear separation of the two basement membranes around blood vessels^15^, suggesting an intense traffic of cells between blood vessels and the spinal cord parenchyma^36^. We monitored here whether these phenomena could also be induced by CM-hADSC (Fig. 9). Animals treated with CM-hADSC had no cell clusters in the spinal cord (Fig. 9B), or separation of vascular basement membranes (Fig. 9E, arrowhead, H, arrow). In contrast, both features were confirmed in the hADSC-treated group (Fig. 9C,F, arrowheads, I, arrows), consistent with our previous findings^14^. Most of the attract endogenous cells in the perivascular region (Fig. 9I) and within the clusters (Fig. 9J) expressed high levels of nestin, while low nestin expression was observed in CM-hADSC (Fig. 9E,H) and DMEM control groups (Fig. 9D,G). Additionally, sequential sections indicate that nestin reactivity correlated with strong expression of NG2, a pericyte marker (Fig. 9J,K). It is interesting that Ki67 + nuclei were found in areas of cells with intense nestin expression, suggesting that this endogenous population could be activated by the treatment with hADSCs. Finally, in order to confirm that pericytes were nestin + , we used double immunostaining for nestin and α-SMA and found extensive co-localization (Fig. 9M), showing that pericytes covering blood vessels in the hADSC-treated animal expressed nestin.

The results of the present study indicate that conditioned media obtained from in vitro cultured hADSC are not effective for treating acute spinal cord injury. The only experimental treatment capable of promoting significant motor recovery was the transplantation of hADSC, followed by the required cellular and molecular modifications of the injured tissues. This was demonstrated by: 1) formation of mature blood vessels, with a high pericyte coverage and better spinal blood barrier integrity, 2) reduction of the cystic cavity and astrogliosis, 3) increased number of regenerative axonal fibres; and 4) modification of the pattern of hADSCs angiogenic factors in response to the spinal cord injury. We conclude that the direct cell-to-cell contact, and/or the local production of the extracellular matrix are essential for hADSC to orchestrate the angiogenic processes, providing the required conditions for the spinal cord regeneration.

## Discussion

Therapeutic use of MSC secretome, such as conditioned media (CM) or purified MSC-derived extracellular vesicles, has become attractive for clinical applications, as they are a more affordable and feasible alternative to MSC transplantation^37^. The major question is whether they can effectively replace stem cell transplantation. hADSCs are reported to stimulate posttraumatic angiogenesis through paracrine secretion of growth factors^14,38^. It is not clear how the vascular reconstruction is coordinated by hADSCs, nor whether the bioactive molecules produced by them in vitro could be used therapeutically for acute injuries, such as stroke or spinal cord injury. Here, we compared the effects of injecting hADSCs or conditioned media obtained from hADSC cultures into the spinal parenchyma after a moderate injury by balloon-compression in rats.

Higher vascularization correlated with functional benefit only in animals treated with hADSCs. On the other hand, vascular density increased in both hADSCs and CM-hADSC therapies as compared to the control, while CM-hADSC presented a significantly higher density than hADSCs in the grey matter both at 1 and 8 WPI. Moreover, vessels with visible BSB tended to be more numerous in animals treated with hADSCs or CM-hADSC than in the DMEM group. These results were consistent with other studies demonstrating that CM from MSCs increases vessels density in experimental models of hindlimb ischemia^39^, peripheral nerve injury^32^ and SCI^40^. ADSC-derived exosomes also stimulate proliferation and differentiation of endothelial cells in vitro^30,41^, and systemic administration of MSCs-exosomes promoted angiogenesis and enhanced neurological recovery in rats after spinal cord injury^42^ or acute ischemic stroke^41^. However, most studies do not assess maturation of the newly formed blood vessels mediated by the MSC supernatants. Here, evidence of vascular immaturity in animals receiving CM-hADSC injection was demonstrated by (1) lack of vascular regression over time, (2) the discontinuous expression of a blood–brain barrier marker, (3) perivascular bleeding and plasma leakage and (4) reduction of pericytes embedded within the vascular wall.

Previous temporal analyses of endogenous vascular repair following SCI demonstrated that a hierarchical regression of secondary vascular branches occurs after 2 WPI, which leads to a decrease in vessel density over time^5,34^. We observed a reduction in vascular density starting as early as 1 WPI, persisting until 8 WPI in animals receiving hADSCs. In the CM-hADSC group, vessels did not regress at 8 WPI, which is indicative of a poorer remodelling. Likewise, there was also no decrease in vessel density in the control group, which seems to be in contrast with previous literature^5,34^. However, it is noteworthy that those reports evaluated vessel densities in areas of newly formed tissue at the centre of the lesion, which, few days before, were occupied by empty cavities. Empty cavities are replenished with growing tissue after a weight-drop contusion within 1 WPI. On the other hand, one week after a 5-min balloon compression, as used here, a large cavity dominates the spinal sections in non-treated animals (Fig. 1A). In our study we quantified blood vessel density in areas contiguous to the cavities, which indicated the onset of angiogenesis at the vicinity of the lesion site^5^. Quantification of SMI-71 + blood vessels also indicated that both cells and their conditioned medium led to an increased vascular density compared to DMEM, whereas SMI-71 labelling in the CM-hADSC group was not continuous. It has been shown in previous studies that discontinuous SMI-71 labelling indicates blood vessel deterioration and breakdown of BSB components, whereas continuous labelling indicates barrier integrity^43^. The patchy pattern of the BSB observed in animals that received CM-hADSC may justify the occurrence of haemorrhagic areas and plasma extravasation. A discontinuous BSB favours leaky blood vessels, contributing to oedema formation, neuronal cell death and damage to white matter tracts^44^. In agreement with results found in our study, the transplantation of hADSCs in the injured spinal cord of dogs reduced occurrence of haemorrhage^17^.

It has also been shown that depletion of pericytes in the spinal cord prevented angiogenesis, enhanced oedema, induced haemorrhage and impaired forelimb functional recovery^7^. A correlation between vascular leakage and reduction of pericytes around the vascular wall was observed in CM-hADSC treated animals. Pericytes play a pivotal role in maintaining vascular integrity and restoring BSB after spinal cord injury^34^. Their adhesion to the vascular wall occurs as soon as blood tubules form and they are essential for vascular stabilization^45^. Previous studies showed that 5 days after SCI there is a loss of vascular coverage by pericytes, increased pericyte detachment from vascular walls, and pericyte morphology changes^46^. Most blood vessels in animals treated with CM-hADSC lacked pericyte coverage, indicating the fragility of new blood vessels. On the other hand, in hADSC-treated animals, pericytes supported the blood vessel tubes that grew at the margin of the lesion-induced cavity. Our results are consistent with those described in the literature, which demonstrate the importance of pericytes in stabilizing new blood vessels after SCI^7^. The depletion of PDGF-Rβ+ pericytes caused severe haemorrhage and impaired recovery of blood vessels^47^.

Pericytes attracted by hADSCs may have acted not only in angiogenesis but also favoured spinal cord regeneration. Pericytes participate in the formation of a healing tissue^8^ and are considered essential for the functional recovery after SCI^45^. It has recently been reported that pericytes may be activated following an ischemic injury to the human brain, identified as ischemia-induced multipotent stem cells (iSCs). This phenotypic change is marked by increased cell proliferation and nestin expression^9^, which was also observed in our study. We had previously shown that the injection of hADSCs into the spinal cord increased the recruitment of cells expressing markers of neural progenitors, such as nestin, β-tubulin III (detected with TUJ-1) and Olig2^15^. We now found evidence that in hADSCs, the cells attracted into the injured spinal cord may well be pericytes, which express α-SMA, NG2, PDGFR-β and nestin. We have also observed that pericytes covering lesion-associated blood vessels expressed nestin in hADSC-treated animals. Nestin has been identified in various cells, including those of neural^48^ and vascular lineages^49^. Further characterization of nestin + pericytes is necessary.

Our study shows that injection of hADSCs into the spinal cord was required for pericytes recruitment. The use of GFP + hADSC was key to understanding the interaction between host cells and transplanted human cells. We confirmed the vascular tropism of human transplanted cells and the lack of direct differentiation into pericytes or endothelial cells, consistent with other studies^13,15,17^. An increase of up to 25 times the number of pericytes in the epicentre of the lesion is expected 3 to 5 days after SCI^8^. There are two subpopulations of pericytes that harbor blood vessels in the spinal cord: subtype A, and subtype B pericytes. The supbpoulation of pericytes A was invariably located abluminal to the B pericyte. Both of them express platelet-derived growth factor receptor (PDGFR) α and β and CD13, but type B pericytes can be distinguished by the expression of desmin and/or alpha smooth muscle actin. In response to spinal cord injury, subtype A pericytes detache from the vascular wall and invade the surrounding tissue. Only subtype A pericytes leaves the vascular niche. They migrate to the center of the injury, surrounded by a layer of astrocytes and proliferate. During this process, they lose their expression of CD13 and PDGFRα, but remain positive for PDGFR β and transiently express αSMA. These αSMA-positive cells, distributed in the parenchyma, participate in the healing of the spinal cord after injury and the ablation of them causes an increase in the area of the cystic cavity^8^. These data corroborate the results found in this article, in which we observed a massive presence of αSMA-positive cells in the spinal parenchyma, attracted by human ADSC, and which were not associated with blood vessels. In addition, it has been demonstrated that type A pericytes occupy the perivascular compartment between the two basement membranes of blood vessels, during the migratory process^8^. It is possible that the perivascular compartment is not only associated with the influx of inflammatory cells^36^, as previously described, but also with the migratory process of type A pericytes, as well as it can also be used as a quick and easy route of mobilization for cells from distant sites. The separation of two basement membranes occurred in the spinal cord of animals receiving hADSCs, but not in the CM-hADSC or DMEM groups, and this is in agreement with our previous study^15^ and supports the hypothesis that the stimulus triggering the phenomenon was hADCS transplantation.

In this study we compared the pro-angiogenic factors secreted by hADSC in vitro and in vivo after SCI and our data suggest that hADSC change their profile of secreted molecules in response to trauma. We observed that while hADSCs produced a broad variety of pro-angiogenic factors in vitro, these molecules were absent in vivo. The CM produced in vitro by hADSCs was rich in VEGF, the major factor that induces vascular sprouting^50^ and revascularization in the spinal cord^51,52^. In contrast, when transplanted to the spinal cord parenchyma, hADSCs preferentially expressed inducers of vascular maturation. In vivo, hADSCs secreted metalloproteinase and plasminogen inhibitors (e.g.: TIMP-1 and Serpin-1), which support blood vessel stabilization^3,53^. Another relevant change was the synthesis of PDGF-AA that only started to be produced by hADSCs after they were injected into the spinal cord, which may explain the absence of pericytes in the blood vessels of those animals that received CM-hADSC. hADSCs are able to generate in vitro three-dimensional vascular structures, and inhibition of PDGF receptors substantially reduced recruitment of α-SMA-expressing pericytes and vessel coverage^54^. PDGF-AA is an important regulator of cell migration and wound healing, and it is a strong stimulator of mesenchymal cells activity^55^. PDGF-AA and PDGF-AB isoforms have been shown to act synergistically during cornea revascularization^56^. Although PDGF-BB or AB were not detected in the spinal cord 24 h after injury, we observed the recruitment of PDGFR-β+ pericytes 7 days after hADSC transplantation, suggesting that paracrine factors may still have been produced by hADSC at later timepoints.

At this point, we cannot exclude the possibility that the absence of some proangiogenic growth factors in the hADSC group resulted from their susceptibility to proteolysis, cell internalization, or to their stable association with the extracellular matrix, interfering with their harvesting for analysis. Growth factors are known to have potentially short half-lives that could account for their absence in the tissue extracts studied here^57^.

The ability of hADSCs to enhance motor recovery of animals, as observed in this study, had also been described previously^10,23^, but the effect of the CM-hADSCs alone had not yet been monitored in SCI. The CM obtained from bone marrow derived-MSC (BM-MSC)^40,58–61^ or from Wharton’s jelly derived-MSC (WJ-MSCs)^61^ was tested in several spinal cord injury models in adult rats and promoted functional recovery^40,58,59,62^, reduction of lesion cavity^58,59,61^, and axonal regeneration^58,60,62,68^, contrasting with the findings presented here. However, in most studies, animals treated with CM were compared only to the control-vehicle group, not to cell transplantation. One study also evaluated the hindlimb motor function of animals treated with WJ-MSC and its respective CM, and only found a significant functional improvement of CM-treated animals in the first week after compression^62^. However, it is important to highlight that MSCs derived from BM, AT or WJ differ in their secretion of neurotrophic, neuroprotective or angiogenic factors^63^.

It has been previously reported that the conditioned medium of mesenchymal cells promotes reduction of lesion area in the chronic phase of SCI in rodents^40,59,62^. We also observed a reduction in cavity areas when we compared animals treated with CM-ADSC and DMEM at 8 WPI (not shown). Nevertheless, cavity reduction was more pronounced in animals that received hADSCs. In contrast to most reports, we did not observe a reduction in glial scar after CM treatment. Instead, we observed that the different treatments led to distinct patterns in GFAP labelling. Astrocytes presented a fibrous shape in DMEM, a densely packed organization with no distinguishable morphology in CM-ADSC and a typical star-shaped morphology associated to BSB in the hADSC group (Supplementary Fig. S4). Animals receiving CM-hADSCs expressed more GFAP around the cystic cavity at 8 weeks than the other groups. Although the possible roles of astrocyte hypertrophy are controversial^64^, many studies demonstrate that they are associated with a microenvironment that is non-permissive to axonal growth^65^. In fact, few TUJ-1 + fibres were able to cross the lesion in CM-hADSC animals. We believe that a controlled astrogliosis, associated with a mature vascular network created the favourable microenvironment for axonal growth, where TUJ-1 + axonal fibres could grow guided by vascular tracks. The gradient of angiogenic growth factors produced in vivo by hADSC could also perform neurotrophic activity. Several factors have this dual function, e.g.; VEGF can stimulate simultaneously tip-cells in vascular growth and axonal growth cone^66^. Laminin present in the vascular basal lamina can also act as a conduit for axonal growth^34^. Previous studies indicate that hADSC produce laminin in the spinal parenchyma, leading to an increase in the numbers of descending regenerating axons in the spinal cord^15^. The correlation between vascular and neural signalling mediated by hADSC still needs to be better studied, but the present results show that they occurred concurrently and were modulated by transplanted hADSC.

## Conclusion

**Figure 10:**
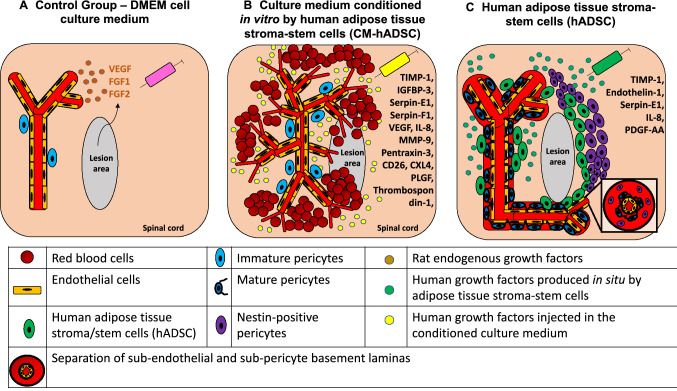
Mesenchymal cell therapy increases angiogenesis in the spinal cord after a moderate balloon-compression injury. The scheme represents the angiogenic process after compression of the spinal cord in control animals (**A**), and in animals treated with culture media conditioned by human adipose tissue-derived stromal/stem cells (CM-hADSC) (**B**) or human adipose tissue-derived stromal/stem cells (hADSC) (**C**). (**A**) In the control animal (DMEM medium) few blood vessels grow spontaneously around the lesion site. (**B**) The injection of the CM-hADSC, rich in pro-angiogenic growth factors, promotes an increase in the number of blood vessels, but they do not have an organized vascular structure, the vascular tubules are very branched, thin, and are not covered with mature pericytes. The lack of vascular maturity results in hemorrhages scattered in the spinal cord and is highlighted by a patchy expression of the BSB marker SMI71. (**C**) Treatment with hADSCs promotes an increase in the vascularization of the spinal cord, with a mature and well-defined vascular architecture. Vessels are characterized by a continuous expression pattern of the BSB marker SMI71. The blood vessels are wider, less branched, and covered by pericytes. The modification of the proteomic profile of the injected cells, mediated by the lesion environment itself, directs the vascular maturation process, attracting pericytes, which stabilize the new blood vessels. Both pericytes and neural precursors that express nestin, which are attracted by hADSCs, migrate through the perivascular space between the two layers of the basal vascular lamina. This promotes functional recovery after SCI.

In the present study, we found that the medium conditioned by hADSC secreta in vitro was sufficient to induce angiogenesis after compressive SCI in rats, but that the physical presence of the cells was required for vessel maturation, attraction of resident pericytes and for regenerative functional improvement. A schematic drawing (Fig. 10) illustrates that both treatments, CM-hADSC (Fig. 10B) and hADSC (Fig. 10C), increase vascular density as compared to spontaneous vascular regrowth observed in control animals (Fig. 10A), but only treatment with hADSCs allows the formation of organised and mature blood vessels. We provided evidence that a change in the secretome of hADSCs within the spinal parenchyma may account for their superior regenerative properties relative to CM-hADSC. Nevertheless, at this point we cannot exclude that other phenomena contribute to explain the necessity of the cells themselves. Living cells offer the possibility of continuous production of factors, which are susceptible to degradation within the tissue. Furthermore, hADSCs could interact directly with endogenous cells or produce ECM molecules to modulate their behaviour. In this regard, we have already shown that hADSCs start producing laminin when injected in the spinal cord^14^, and laminin has been shown to serve as a guiding cue for both regenerating axons^15,67^ and pericytes contributing for vessel maturation^6^. The study presented here underlines the importance of further investigation towards understanding the interactions of hADSC, pericytes and neural precursors in the scenario of SCI, since transplantation of hADSCs represents an encouraging new perspective for the treatment of CNS injuries.

## Methods

### Animals

We used 40 adult female Sprague–Dawley rats (200–250 g), bred at the animal facility of the Federal University of Rio de Janeiro. Rats were kept in 12 h light/dark cycles, with free access to food and water in an isolated animal room. They were submitted to spinal cord compression and subdivided in: (I) the cell treated group (hADSC group), (II) the conditioned medium treated group (CM-hADSC group) and (III) the vehicle control (DMEM group). Sham operated animals were also included for functional test.

### Human cells

Fragments of subcutaneous adipose tissue and lipoaspirates were obtained from the abdominal region of patients undergoing plastic surgery at the Clementino Fraga Filho University Hospital (HUCFF), Federal University of Rio de Janeiro, Brazil.

### Plasmid construct and lentivirus production

The pLL3.7 plasmid^68^ was provided by Professor Guido Lenz (UFRGS – Porto Alegre, Brazil). Lentiviral vectors were produced as previously described^69^.

### Isolation and transduction of hADSCs

hADSCs were obtained as previously described^70^. Lentivirus transduction was done by addition of 1 mL lentiviral vector stock with polybrene, 8 mg/mL, and 7 mL DMEM. Cells were incubated at 5% CO_2_ and 37 °C overnight, followed by fresh DMEM with 10% fetal bovine serum, until they were prepared for the injection. The typical multiplicity of infection (MOI) used for ADSC transduction ranged from 10 to 50. Transduced cells were trypsinized, fixed with 4% paraformaldehyde in phosphate-buffered saline, and GFP expression was analysed by flow cytometry (BD FACScalibur Flow Cytometer; Becton Dickinson) using the CellQuest software. We found that 60–70% of hADSCs were GFP+. Different batches of transduced cells were routinely checked for quality, potential contamination or unexpected properties. They were stable and we found no evidence of significant loss or decease of the GFP+ population upon cultivation.

### Preparation of the conditioned medium from hADSC (CM-hADSC)

The conditioned medium of the hADSCs (CM-hADSC) was obtained after expansion of the hADSCs in DMEM with 10% FBS. When reaching 90% confluence, cells were washed to remove the FBS, and the medium was replaced by DMEM/F12 without serum. After one week, the supernatant was harvested, centrifuged to remove cell debris and stored at − 20 °C until use. Immediately after supernatant harvesting, cells remaining in flasks were trypsinized and counted using trypan blue dye (0.4% in PBS). Cell viability was above 90%.

### Surgical procedures

Animals were anesthetized and subjected to dorsal laminectomy. For the compression injury, the T7 vertebra was removed and a 2-French Fogarty catheter was inserted into the dorsal epidural space, caudally to T8–T9 spinal level. The balloon was inflated with 15 µL water for 5 min. Rats were kept anesthetized for 30 min before application of acute experimental treatments. Soft tissue and skin were sutured in anatomical layers. All animals had their eyes lubricated during surgery, received subcutaneous 10 ml injections of Ringer solution for hydration after surgery, and were treated with gentamicin sulphate for 3 days to avoid urinary infection. No immunosuppression treatments were used. The only drugs administered were pain relievers in the first 3 days after compression. The bladder was manually emptied until spontaneous function was re-established, typically 3 days after injury.

### Spinal cord injections

Ten microliters of cell suspensions, CM or vehicle (DMEM) were stereotaxically injected into the parenchyma of the lesioned spinal cord, using a 10 μl Hamilton syringe. Injections were made in the T7 segment, which is the region exposed by the laminectomy. The catheter used to inflict the lesion slides 1 cm caudally to reach the T8/9 segment, which is the centre of the lesion. Therefore, cells transplanted at T7 reach the spinal cord at 1 cm rostrally of the lesion epicentre. Upon reaching 80–90% confluence, hADSCs were trypsinized, counted, centrifuged and ressuspended in a small volume as to give 5 × 10^5^ cells in 10 μL DMEM.

### Histology and Immunofluorescence

Rats were anesthetized and perfused transcardially with 4% paraformaldehyde in 0.1 M phosphate buffer (pH 7.4). Spinal cords were removed, and a 1 cm segment between T7–T10 levels was harvested. For immunofluorescence analysis, segments were cut in the transversal or horizontal planes with a vibratome and collected serially onto gelatine-coated glass slides.

Prior to immunolabeling, slides were extensively washed with PBS, permeabilised with 0.3% Triton-X-100, blocked with 10% bovine serum albumin or normal goat serum, and incubated overnight at 4 °C with primary antibodies.

The following mouse antibodies were used: anti-GFAP (1:500; Merck Millipore); anti-RECA-1 (1:100; AbD Serotec), anti-alpha smooth muscle actin (1:400; Sigma), anti-class III β-tubulin (TUJ-1; 1:500; R&D), SMI-71 (1:200; Biolegend). The following rabbit antibodies were used: anti-PDGFRbeta (1:200; Abcam), anti-Ki67 (1:100; Abcam); anti-nestin (1:500; Merck Millipore; 1:100; Santa Cruz); anti-NG2 (1:200; Merck Millipore); pan-laminin (polyclonal antibody raised against EHS laminin 1:50; Merck Millipore), anti-GFAP (1:400; Dako). We used Isolectin IB4 Biotin Conjugated Antibody (1:200; Vector B-1205), and Dapi (1:4,000; Merck Millipore). After rinsing, sections were incubated for 2 h at room temperature with fluorescent dye-conjugated goat anti-mouse or anti-rabbit IgGs (Cy3-conjugated from Merck Millipore, Alexa 633, Alexa 594 and Alexa 488-conjugated from ThermoFisher). In order to confirm blood leakage, we incubated spinal cord sections with fluorescent anti-rat IgG (ThermoFisher), without primary antibodies incubation. After an additional wash with PBS and distilled water, slides were mounted with n-propyl gallate (Merck Millipore). In negative controls the primary antibodies were omitted.

### Image analyses

Tissue and cells were analysed in a TE200 fluorescence inverted microscope (Nikon), with standard DAPI/FITC/TRITC filters coupled to a colour CCD camera (Evolution, Media Cybernetics) and using the software Image Pro Plus 6.0 for image processing (Media Cybernetics Inc.). Images were alternatively acquired with a TCS-SP5 confocal microscope (Leica).

Quantitative analyses of vascular densities at perilesional areas were performed 1 and 8 WPI. The sections containing the largest cavities for each animal (between 600 and 750 μm deep from the dorsal surface) were identified after immunostaining with anti-GFAP and DAPI of 1 out of each 3 histological sections. The consecutive sections were labelled with RECA-1 and vessels counted within the preserved tissue around the cavities. For quantification of blood vessels bearing BSB, we used SMI-71 to label sections corresponding to 450–600 μm deep from the dorsal surface. We evaluated three animals of each experimental group, in 4–8 distinct fields, photographed at 20 × in the conventional fluorescence microscope (for RECA-1 immunostaining sections) or confocal microscopy (for SMI-71 immunostaining sections). Blood vessel lengths were calculated using Image Pro Plus 6.0 software (Media Cybernetics, Inc.) and AngioTool software as previously described^71^.

### Angiogenesis related protein expression profile

To analyse angiogenesis-related factors we used a species-specific human protein detection assay. We compared two samples: hADSCs in vitro, *i.e.* factors released by cells in the culture supernatant and hADSCs in vivo, *i.e.* the factors produced by cells after injection into the lesioned spinal cord. Samples were collected after 24 h of either cultivation or injection into the injured spinal cord. In the latter case, animals were euthanatized in a CO_2_ chamber, the spinal cords were dissected in ice-cold PBS and1 cm long segments bearing the lesion were removed. The nervous tissue was homogenized in a Potter-type homogenizer in RIPA buffer (pH 7.0) with a cocktail of protease inhibitors and the obtained suspensions were centrifuged at 3500 × *g* for 20 min at 4 °C. The supernatants were collected and stored at − 20 °C until the assay was performed. Samples of three animals from each experimental group were pooled for the analysis. Samples of secreted factors produced in vitro were processed simultaneously with the tissue samples obtained in vivo from the experimental animals. Expression of angiogenesis-related proteins by hADSCs in vitro and from tissues of rats that had received injections of hADSC (“CM-hADSCs in vivo”) or of the conditioned medium of cultured hADSCs (“CM-hADSCs in vivo”) were normalized for total protein content and assessed by a semi-quantitative assay using a proteomic profile kit for human angiogenic factors (ARY007, R&D Systems), following the manufacturer's instructions. Data are presented as relative ODs, using the absorbance of the positive control of the assay as 1.

### Behavioural assessment

Locomotor activity was evaluated using the open-field walking test, BBB^33^. Each animal was allowed to move freely inside a circular plastic tray for 5 min, and two independent examiners who were blinded to the experiment attributed scores. The final score of each animal was the mean value of the two examiners.

### Statistics

Comparisons between different experimental groups were performed using GraphPad Prism version 5.00 for Windows (GraphPad Software, San Diego, USA) to analyse the variance (ANOVA) with post-hoc Kruskal–Wallis test for multiple comparisons (BBB assays). To evaluate the differences between the BBB test scores, the Mann–Whitney test suitable for non-parametric values was used. To evaluate the differences in the expression of angiogenesis-related proteins, the one-way-ANOVA with a Dunnett post-test was used, comparing all the experimental columns with the control. In all tests, the assumed confidence interval was 95%.

### Ethical approval and informed consent

All experimental procedures adhered to the guidelines of the American National Institute of Health and of the Brazilian COBEA, and they were approved by the Animal Welfare Committee of the Federal University of Rio de Janeiro / Centre of Health Sciences [DAHEICB 041]. All procedures involving human samples were approved by the Investigational Review Board at the University Hospital HUCFF (protocol numbers 043/09 and 088/04). Adipose tissue and lipoaspirates were collected after the patients signed a written informed consent.

## Supplementary information

Supplementary Information.

## Data Availability

All supporting data is available for the evaluation for Editorial Board Members and referees.
