# Supplementary material for: Human mesenchymal stromal/stem cells recruit resident pericytes and induce blood vessels maturation to repair experimental spinal cord injury in rats
Source: Sci Rep. 2020 Nov 11;10:19604. doi: 10.1038/s41598-020-76290-0 (PMC7658254; doi:10.1038/s41598-020-76290-0)
Supplement: Supplementary file 1 — Supplementary Information. [file 41598_2020_76290_MOESM1_ESM.pdf]

Human mesenchymal stromal/stem cells recruit resident pericytes and induce blood vessels maturation to repair experimental spinal cord injury in rats

AUTHORS AFFILIATIONS AND CONTACT INFORMATION

Karla Menezes*[1,2]: Menezes, K.; karlamenezess@gmail.com; https://orcid.org/0000-0002-4343-5587.

Barbara Gomes Rosa[1]: Barbara GR; barbara.gomes.rosa@gmail.com; https://orcid.org/0000-0002-40265586

Catarina Freitas[1]: Freitas C; freitas.catarina@gmail.com; https://orcid.org/0000-0002-3393-5326

Aline Silva da Cruz[3]: Cruz AS; acruz@peb.ufrj.br; https://orcid.org/0000-0001-9976-1851.

Raphael de Siqueira Santos[4]: Siqueira-Santos, R; rss_uff@yahoo.com.br https://orcid.org/0000-0001--8942-0850

Marcos Assis Nascimento[4]: Nascimento, M.A.; marcos.assis@gmail.com; https://orcid.org/0000-0002-9830-9801.

Daiana Vieira Lopes Alves[5]: Lopes-Alves, D.V; daianalopes8@gmail.com; orcid.org/0000-0002-1936-1952.

Martin Bonamino[6]: Bonamino, M.; mbonamino@inca.gov.br; http://orcid.org/0000-0002-4416-5822

Maria Isabel Rossi[1]: Rossi, M.I. idrossi@hucff.ufrj.br; http://orcid.org/0000-0003-3432-8453.

Radovan Borojevic[1,2]: Borojevic, R. rrborojevic@gmail.com; http://orcid.org/0000-0002-2393-7280.

Tatiana Coelho-Sampaio[1]: Coelho-Sampaio, T.; tcsampaio@histo.ufrj.br; http://orcid.org/0000-0002-3020-9580.

1. Institute of Biomedical Sciences, Federal University of Rio de Janeiro. Ave. Carlos Chagas Filho 373, Sala B1-011, Cidade Universitária, Ilha do Fundão, Rio de Janeiro, RJ, 21941-590, Brazil.
2. Center for Regenerative Medicine, Petrópolis Faculty of Medicine, Av. Barão do Rio Branco 1003, Centro, Petrópolis RJ 25680-120, Brazil.
3. Alberto Luiz Coimbra Institute for Graduate Studies and Engineering Research (COPPE), Federal University of Rio de Janeiro, Ave. Carlos Chagas Filho 373, Sala B1-011, Cidade Universitária, Ilha do Fundão, Rio de Janeiro, RJ, 21941-590, Brazil
4. Institute of Biophysics Carlos Chagas Filho, Federal University of Rio de Janeiro. Ave. Carlos Chagas Filho 373, Sala G2-053, Cidade Universitária, Ilha do Fundão, Rio de Janeiro, RJ, 21941-590, Brazil.
5. Institute for Biodiversity and Sustainability - NUPEM, Federal University of Rio de Janeiro; Ave. São José do Barreto s/n - São José do Barreto, Macaé, RJ, 27965-045, Brazil.
6. National Institute of Cancer, Rio de Janeiro, Marquês de Pombal, Centro, Rio de Janeiro, RJ, 20230-240, Brazil.

* Correspondence should be addressed to Karla Menezes: karlamenezess@gmail.com

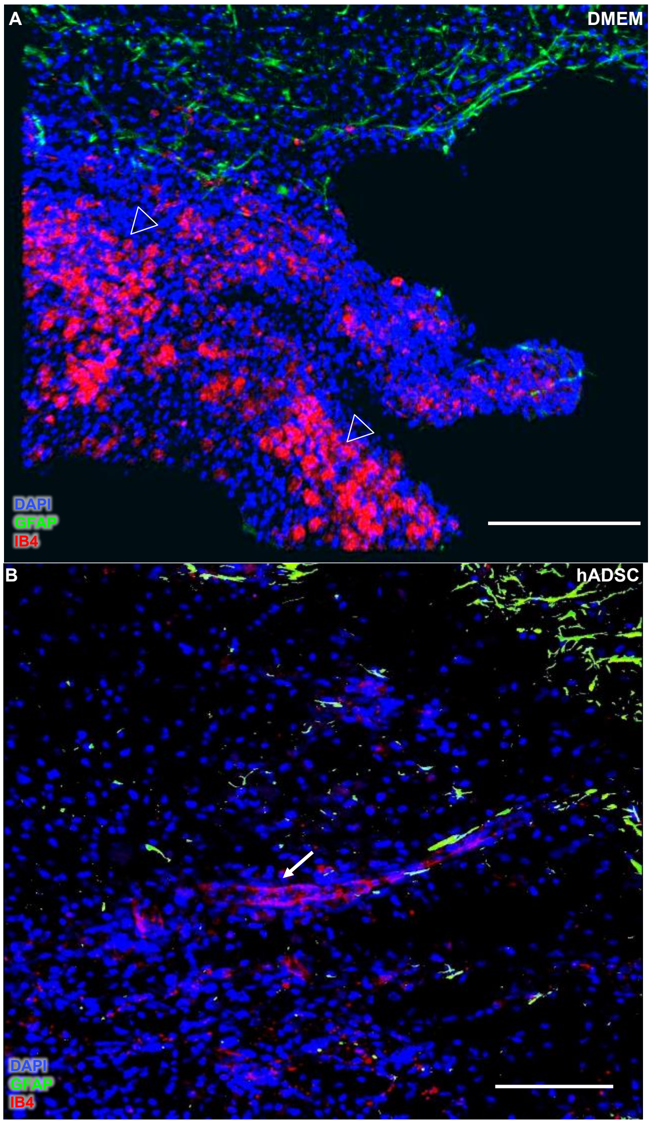

Supplementary Figure 1. **Morphological difference between blood vessels and phagocytic cells identified with the IB4 antibody in rat spinal cord injury**. Confocal images of the spinal cord (horizontal sections) of rats treated with DMEM (A) and hADSC (B), 1 week after SCI, labelled with Isolectin IB4 (red), anti-GFAP antibody (green), and DAPI (blue), to identify blood vessels and phagocytic cells, astrocytes and nuclei. Note that endothelial cells can be visualized as a tubular shape and phagocytic cells as rounded cells. White arrows indicate blood vessels and arrowheads with white outline indicate phagocytic cells. Scalebar: A-B: 200 μm

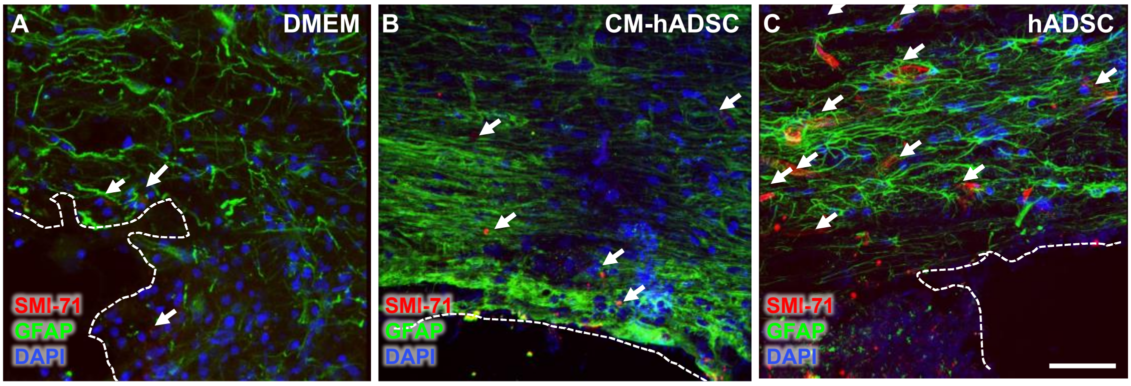

Supplementary Figure 2: hADSC increases the presence of spinal cord blood barrier in the late spinal cord injury A, B, C) Confocal images of the spinal cord (horizontal sections) of rats treated with DMEM (A), CM-hADSC (B) and hADSC (C), showing blood vessels identified with a specific blood brain barrier antibody, anti-SMI-71 (red), and astrocytes, identified by anti-GFAP (green), and DAPI-stained nuclei (blue). White dotted lines delimit cavity areas, and white arrows indicate blood vessels (anti-SMI-71 positive). Scalebar: A-C: 50 μm.

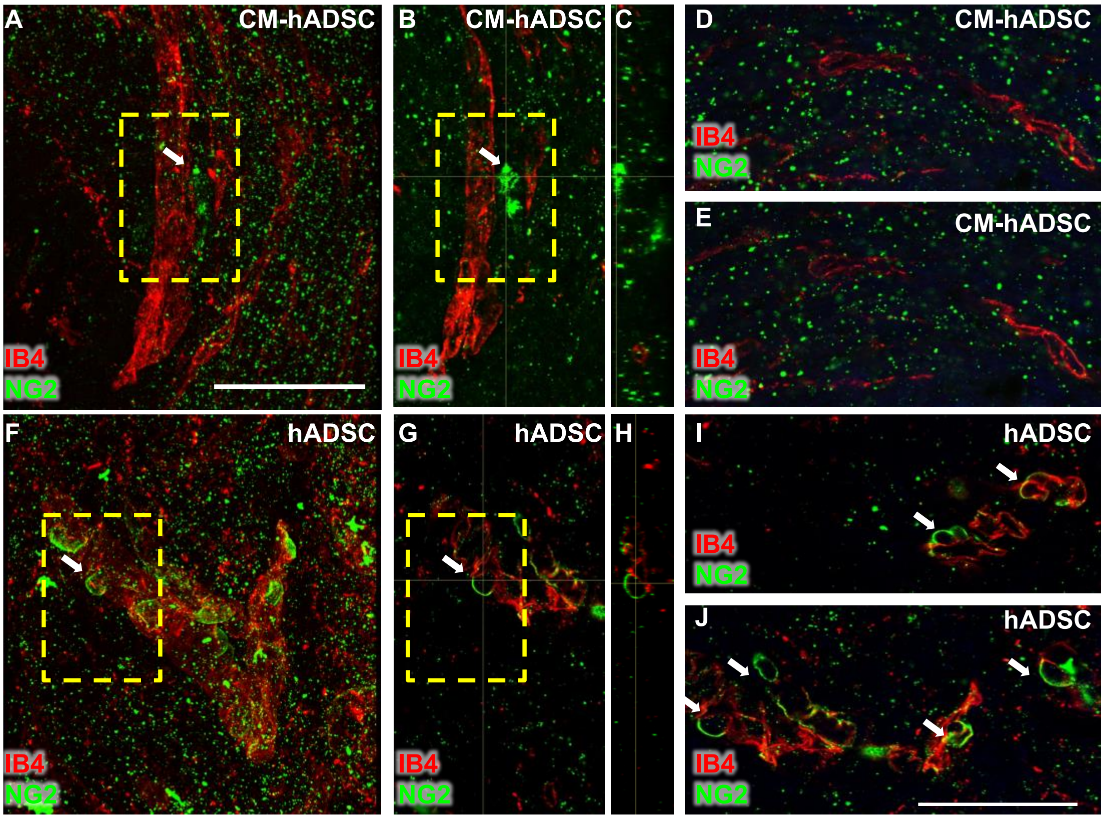

Supplementary Figure 3: Most blood capillaries in the CM-hADSC group did not present pericytes and, when present, they did not intimately contact the vascular wall. A-J) Confocal images of horizontal sections of the spinal cord were analyzed at 1 WPI of CM-hADSC (A-E) and hADSC groups (F-J). A and D represent top views of 3D reconstructions and B, C, G and H correspond to orthogonal projections of confocal stacks. D, E, I and J correspond to optical slices. In order to analyze the maturity of blood vessels, histological sections were immuno-labelled with anti-NG2 antibody (green), isolectin-4 (IB4) (red) and DAPI (blue) to identify pericytes, endothelial cells and nuclei, respectively. The white arrows mark pericytes. In CM-hADSC treated animals (A-E) pericytes were rare and when present they were distant from the vascular wall. On the other hand, in hADSC animals (F-J) pericytes were distributed along the extension of the whole vascular wall, flattened and in close contact with the vascular basement membrane. Scalebar: A-J: 50 μm.

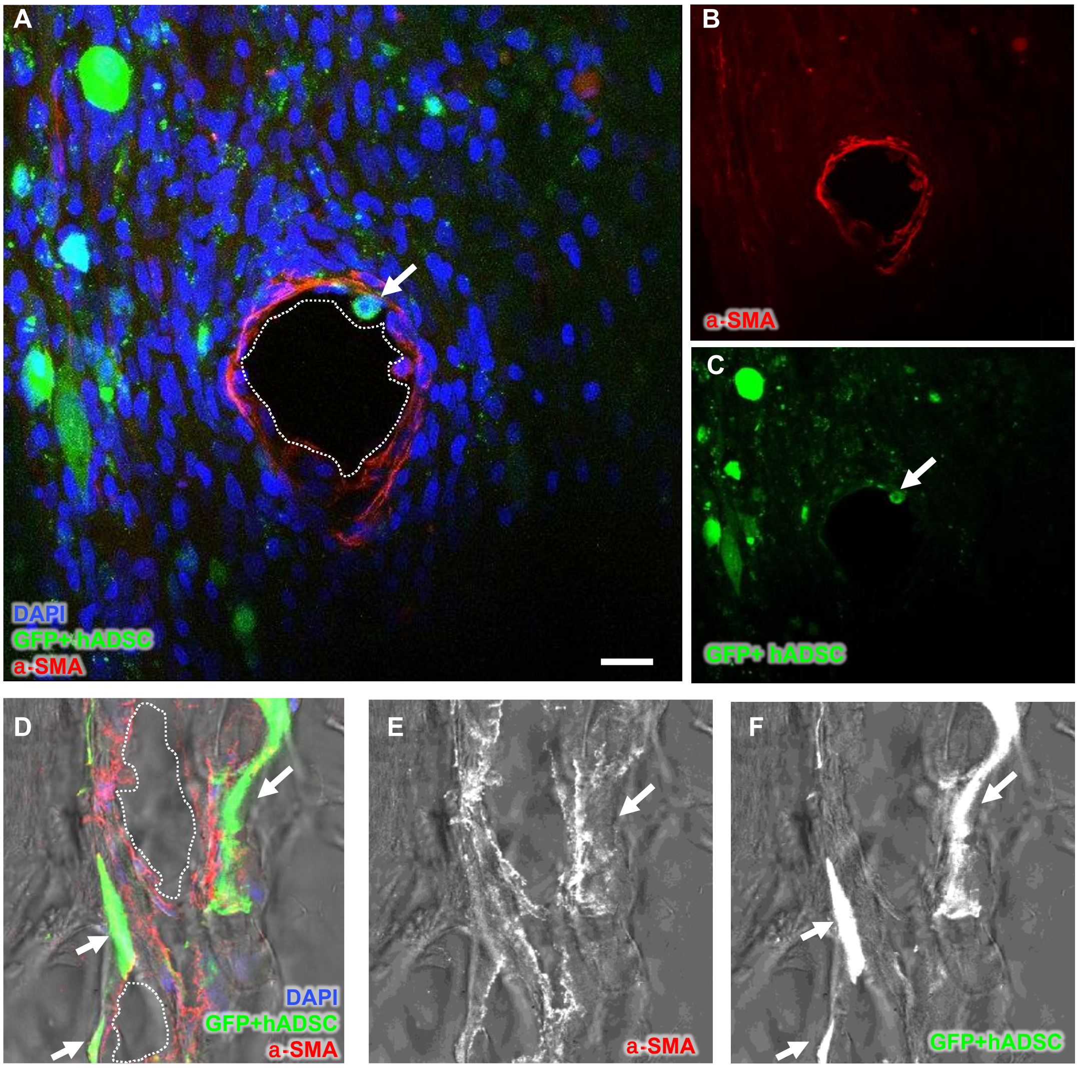

Supplementary Figure 4: hADSC rarely expressed alpha smooth muscle actin (α−SMA), despite being in contact with vascular pericytes. A-F) Confocal images of horizontal sections of the spinal cord, 1WPI, with GFP-transduced hADSCs (green). The nervous tissue was immuno-labelled with alpha smooth muscle actin antibody (anti-α−SMA) and DAPI to identify pericytes (red) and counterstained with DAPI for cell nuclei (blue). We observed that GFP+hADSCs were distributed predominantly around the rat's blood vessels. It is even possible to observed hADSC in close contact with pericytes from the vascular wall, which express α−SMA (red). In the images in D, E, F, the histological sections were visualized in confocal microscopy also with bright field. Note that GFP+hADSCs rarely express α-SMA. White dotted lines delimit blood vessel lumen; white arrows indicate GFP+hADSCs that did not express α-SMA. Scalebar: A: 20 μm.
